# Effect of Arthritic Synovial Fluids on the Expression of Immunomodulatory Factors by Mesenchymal Stem Cells: An Explorative *in vitro* Study

**DOI:** 10.3389/fimmu.2012.00231

**Published:** 2012-08-02

**Authors:** Maarten J. C. Leijs, Gerben M. van Buul, Erik Lubberts, Pieter K. Bos, Jan A. N. Verhaar, Martin J. Hoogduijn, Gerjo J. V. M. van Osch

**Affiliations:** ^1^Department of Orthopaedics, Erasmus MC University Medical CenterRotterdam, The Netherlands; ^2^Department of Radiology, Erasmus MC University Medical CenterRotterdam, The Netherlands; ^3^Department of Rheumatology, Erasmus MC University Medical CenterRotterdam, The Netherlands; ^4^Department of Internal Medicine, Erasmus MC University Medical CenterRotterdam, The Netherlands; ^5^Department of Otorhinolaryngology, Erasmus MC University Medical CenterRotterdam, The Netherlands

**Keywords:** MSC, osteoarthritis, rheumatoid arthritis, synovial fluid, immunomodulation

## Abstract

**Background:** In diseased joints, the catabolic environment results in progressive joint damage. Mesenchymal stem cells (MSCs) can have immunomodulatory effects by secreting anti-inflammatory factors. To exert these effects, MSCs need to be triggered by pro-inflammatory cytokines. To explore the potential of MSCs as a treatment for diseased joints, we studied the effect of synovial fluid (SF) from donors with different joint diseases and donors without joint pathology on the immunomodulatory capacities of human MSCs *in vitro*. We hypothesized that SF of diseased joints influences the immunomodulatory effects of MSCs. **Materials and Methods:** MSCs were cultured in medium with SF of six osteoarthritis (OA) or six rheumatoid arthritis (RA) donors and three donors without joint pathology were used as control. Gene expressions of IL-6, HGF, TNFa, TGFb1, and indoleamine 2,3-dioxygenase (IDO) were analyzed. l-kynurenine concentration in conditioned medium (CM) by MSCs with SF was determined as a measure of IDO activity by MSCs. Furthermore, the effect of CM with SF on proliferation of activated lymphocytes was analyzed. **Results:** Addition of SF significantly up-regulated the mRNA expression of IL-6 and IDO in MSCs. SF(OA) induced significantly higher expression of IDO than SF(control), although no difference in IDO activity of the MSCs could be shown with a l-kynurenine assay. Medium conditioned by MSCs with SF(OA or RA) suppressed activated lymphocyte proliferation *in vitro* more than medium conditioned by MSCs without SF or with SF(control). **Discussion:** SF can influence the expression of genes involved in immunomodulation by MSCs and the effect on lymphocyte proliferation. We found indications for disease-specific differences between SFs but the variation between donors, even within one disease group was high. These data warrant further research to examine the potential application of MSC therapy in arthritic joints.

## Introduction

Osteoarthritis (OA) and rheumatoid arthritis (RA) are high prevalent forms of arthritis. OA is mainly characterized by progressive functional loss and cartilage degeneration. Main factors involved in cartilage degeneration are a variety of matrix degrading enzymes and pro-inflammatory cytokines (Goldring, 2000; Goldring and Marcu, 2009). It is possible to treat the symptoms of OA with lifestyle changes, analgesics, non-steroidal anti-inflammatory drugs (NSAIDs), or intra-articular injections with corticosteroids or hyaluronic acid and the ultimate treatment for end stage OA is joint replacement. A treatment to cure OA, however, is still not available. RA is an auto-immune disease initiated by immune complexes that together with cytokines, complement, and metalloproteinases (Weissmann, 2006) cause an inflammatory and catabolic environment in the joint (Goldring and Marcu, 2009). It is a systemic disease characterized by persistent synovitis, systemic inflammation, and auto-antibodies which eventually cause joint damage with progressive cartilage degeneration and bone alterations. There is a wide range of therapeutic options for RA like analgesics, NSAIDs, disease-modifying anti rheumatic drugs (DMARDs), and biologicals (Lee and Weinblatt, 2001; Scott et al., 2010). However, to date there is no treatment available to cure RA.

Human mesenchymal stem cells (MSCs), the progenitors of connective tissue cells, are able to differentiate into different cell types including chondrocytes (Caplan, 1991, 1994; Solchaga et al., 2004; Caplan and Dennis, 2006). This has attracted the interest of many people working in the area of cartilage repair. Besides the ability to reconstruct tissues, MSCs also have the ability to modulate the environment by secreting many immunomodulating and trophic factors like cytokines, chemokines, and growth factors (Deans and Moseley, 2000; Minguell et al., 2001; Kim et al., 2005; Caplan and Dennis, 2006; Chen et al., 2006; Schinkothe et al., 2008; Hoogduijn et al., 2010; Meisel et al., 2011). These factors have potent immunomodulatory capacity as demonstrated *in vitro* by inhibition of T-lymphocyte proliferation after adding MSCs in mixed lymphocyte reactions (Hoogduijn et al., 2010; Landgraf et al., 2011). MSCs also inhibit the antibody production of B lymphocytes and inhibit the generation and function of antigen presenting cells (Sze et al., 2007; Chen et al., 2008; Hoogduijn et al., 2010). The stimulation of MSC by pro-inflammatory cytokines like TNFa and IFNg strongly enhances the immunosuppressive function of MSCs (Klyushnenkova et al., 2005; Schinkothe et al., 2008; Siegel et al., 2009; Eggenhofer et al., 2010; Hoogduijn et al., 2010).

In a healthy joint environment, a balance exists between an anabolic and catabolic state. In a situation of inflammation or chronic damage, i.e., OA or RA, the environment becomes more catabolic (Findlay and Haynes, 2005; Goldring and Marcu, 2009). All joint tissues are exposed to synovial fluid (SF) and in OA and RA inflammatory factors are secreted into the SF. The aim of the present study was to investigate whether SF of donors with OA, RA, or no joint pathology triggers MSCs to become immunomodulatory. Since inflammation plays a large role in RA and OA, we hypothesized that MSCs will be triggered to become immunomodulatory. We explored this by studying the effect of SF of OA and RA patients as well as SF of non-pathological(control) donors on MSCs. Our hypothesis was that MSCs conditioned in SF(RA) will express a large anti-inflammatory effect compared to SF(control) due to the high inflammation state of RA patients and MSCs conditioned with SF(OA) will express a mild anti-inflammatory effect compared to SF(control) as a reaction to a less inflamed environment in joints of OA patients.

We evaluated the effect of SF on expression of genes of MSCs for immunomodulatory factors. Furthermore, we performed a functional assay to study the capacity of factors secreted by MSCs in response of SF to inhibit proliferation of activated lymphocytes.

## Materials and Methods

### Synovial fluids

Fifteen SF samples were obtained from six OA patients, six RA patients, and three donors without any joint pathology. SFs(OA) were obtained from patients undergoing total knee replacement surgery. All patients implicitly consented to the use of these fluids for scientific research (with approval by Erasmus MC medical ethical committee protocol # MEC-2004-322). SFs(RA) were obtained from RA patients with active inflammation of the knee during consultation at the rheumatology outpatient clinic (with approval by Erasmus MS medical ethical committee protocol # MEC-236.904-2003-255). SFs(control) were purchased from SF donors without joint diseases, post mortem within 24 h of death (Articular Engineering, Northbrook, IL, USA). After aspiration, all SF samples from the joints of all donors were centrifuged to remove debris. Supernatant was stored at −80°C.

To evaluate the inflammatory aspects of the different SFs we did amplified enzyme linked immunosorbent assays (ELISA) to quantify cytokines IL-6, TNFa (R&D Systems, Minneapolis, MN, USA), and IFNg (Invitrogen, Carlsbad, CA, USA). Measurements of IL-6, TNFa, and IFNg were performed in duplicate. All SFs were treated with 1:3 hyaluronidase (1000 U/ml PBS, 10 min at 37°C) prior to ELISA measurements. ELISAs were carried out according to the manufacturer’s instructions by means of a multilabel plate reader (VersaMax™, Molecular Devices, Sunnyvale, CA, USA).

### MSC isolation

Mesenchymal stem cells were isolated from heparinized femoral-shaft marrow aspirate of patients undergoing total hip arthroplasty (with informed consent after approval by Erasmus MC medical ethical committee protocol # MEC-2004-142). About 5–10 ml marrow was harvested with a sterile Jamshidi needle into sterile 10 ml syringes containing 0.5 ml of heparin (1000 U/ml). About 30–100 × 10^6^ mononuclear cells were plated in a T175 flask in 25 ml expansion medium (Dulbecco’s Modified Eagle Medium (DMEM) low glucose (Invitrogen, Carlsbad, CA, USA) containing 15% heat inactivated fetal calf serum (Lonza, Verviers, Belgium, selected batch), 1.5 μg/ml fungizone (All Invitrogen, Carlsbad, CA, USA), 50 μg/ml gentamicin (Invitrogen, Carlsbad, CA, USA), 1 ng/ml fibroblast growth factor-2 (Instruchemie B.V., Delfzijl, The Netherlands), and 0.1 mM of l-ascorbic acid 2-phosphate (vitamin C; Sigma, St. Louis, MO, USA). After 24 h, non-adherent cells and erythrocytes were removed by washing three times with 2% FCS in 1× PBS (Invitrogen, Carlsbad, CA, USA). Remaining adherent cells were cultured in expansion medium at 37°C and 5% carbon dioxide (CO_2_). Expansion media were renewed twice a week. At subconfluent cells were trypsinized with a 0.25% trypsin solution containing 0.01% EDTA (Invitrogen, Carlsbad, CA, USA) and plated at a density of 2300 cells/cm^2^.

### MSC culture with SF

Cryopreserved MSCs of passage two were used for the experiments. After thawing, MSCs were seeded in a T175 flask at a density of 2300 cells/cm^2^, expanded for one passage and subsequently plated in six well plates at a density of 4000 cells/cm^2^ for the experimental conditions. At 70% confluence the existing medium was discarded and the cells were washed three times using PBS (Invitrogen, Carlsbad, CA, USA). Subsequently 0.8 ml of DMEM low glucose containing 9 μg/ml fungizone and 50 μg/ml gentamicin, was applied per well. The different SFs(OA, RA, and control) were added in triplicates to the media in a concentration of 20%. In preliminary tests MSCs were cultured in 0, 10, or 25% SF of four OA donors, Gene expression was not significantly different in 10 and 25% SF. Based on this and taking into account the availability of the SF (from SF(control) we obtained maximal 1 ml per donor) we decided to use 20% SF for all further experiments. All conditions contained a total concentration of 1% ITS (BD Bioscience, Bedford, MA, USA). Nine wells with only medium plus 1% ITS were used as negative controls for unstimulated MSCs. After 48 h of incubation, MSCs were harvested for gene expression analyses and the conditioned medium (CM) was harvested and stored at −80°C.

### Gene expression analysis

After 48 h of incubation total RNA from MSCs was isolated using RNeasy^®^ microkit (Qiagen, Hilden, Germany) with RNeasy MinElute spin columns. After quantification of nucleic acids by spectrophotometry (NanoDrop 2000, Thermo Scientific, Isogen Life Science, IJsselstein, The Netherlands) the RNA was reverse transcribed using a First Strand cDNA Synthesis kit (RevertAid™; MBI Fermentas, St. Leon-Rot, Germany). Amplifications were performed as 20 μl reactions with real-time PCR. Thermocycler conditions comprised an initial holding at 95°C for 10 min, followed by one step at 95°C for 15 s and 60°C for 60 s for 40 cycles. A dissociation stage was added at the end using 95°C for 15 s, 60°C for 20 s, and 95°C for 15 s. For UBC, IL-6, HGF, TNF-α, qPCR™ Mastermix Plus for SYBR^®^ Green I (Eurogentec, Nederland B.V., Maastricht, The Netherlands) was used. For GAPDH, HPRT, IDO, and TGF-β1 TaqMan Master Mix (ABI, Branchburg, NJ, USA) was used. Sets of primers and probes used in this study: GAPDH (NM_002046.3) Fw: ATGGGGAAGGTGAAGGTCG Rv: TAAAAGCAGCCCTGGTGACC Probe: Fam-CGCCCAATACGACCAAATCCGTTGAC; HPRT (NM_000194.2) Fw: TATGGACAGGACTGAACGTCTTG Rv: CACACAGAGGGCTACAATGTG Probe: Fam-AGATGTGATGAAGGAGATGGGAGGCCA; UBC (NM_021009.5) Fw: ATTTGGGTCGCGGTTCTTG Rv: TGCCTTGACATTCTCGATGGT; IL-6 (NM_000600.3) Fw: TCGAGCCCACCGGGAACGAA Rv: GCAGGGAAGGCAGCAGGCAA; HGF (NM_000601.4) Fw: GGCTGGGGCTACACTGGATTG Rv: CCACCATAATCCCCCTCACAT; TNF-aplha (NM_000594.2) Fw: GCCGCATCGCCGTCTCCTAC Rv: AGCGCTGAGTCGGTCACCCT; TGF-beta1 (NM_000660.4) Fw: GTGACAGCAGGGATAACACACTG Rv: CATGAATGGTGGCCAGGTC Probe: Fam-ACATCAACGGGTTCACTACCGGC. IDO was detected using a taqman assay on demand (Applied Biosystems, Capelle a/d IJssel, The Netherlands) of which the primer sequence is not known to us. Data were collected and quantitatively analyzed on an ABI Prism 7000 Sequence Detection System (SDS) with SDS software, version 1.2.3 (Applied Biosystems, Capelle a/d IJssel, The Netherlands). Gene expressions of the cytokines and IDO in MSCs were calculated by cycle threshold (CT) values. CT values of 36 and higher were considered as non-expressed and set to 100 for further calculations. The CT values of the housekeeper genes GAPDH, HPRT, and UBC were averaged by using geometric averaging of every sample. This average is the best keeper index (BKI) for every single sample. All separate CT values were corrected to the BKI by using the 2^−ΔCT^ formula.

### l-kynurenin assay

In order to evaluate whether SF influenced IDO activity in MSCs, we measured the concentration of l-kynurenine in the CM and SFs. To correct for possible l-kynurenine in SF, the SFs were diluted in the same concentration and the same media as the CM and values were subtracted from the CM values. Values of one of the OA donors could not be used since no remaining SF was available for correction. Thirty percent trichloroacetic acid was added to the samples in a 1:3 ratio and after 30 min incubation at 50°C the samples were centrifuged at 12000 rpm for 5 min. Supernatant of all conditions were diluted 1:1 in Ehrlich reagent (200 μg 4-dimethylaminobenzaldehyde (Sigma, St. Louis, MO, USA) in 10 ml of glacial acetic acid) in duplicate in a 96-wells flat bottom plate and absorbance was determined at 490 nm in a multilabel plate reader (VersaMax™, Molecular Devices, Sunnyvale, CA, USA). l-kynurenine (Sigma, St. Louis, MO, USA) was used as standard.

### PBMC proliferation assay

Peripheral blood mononuclear cells (PBMCs) were isolated from buffy coats (Sanquin, Rotterdam, The Netherlands) of healthy volunteers using Ficoll-Paque™ Plus (GE Healthcare, Uppsala, Sweden) separation and stored at −135°C until use. PBMCs were thawed and centrifuged at 2000 rpm for 5 min. Viable cells were counted using trypan blue exclusion test. PBMCs were seeded in alpha-modified Minimum Essential Medium (aMEM; Invitrogen, Carlsbad, CA, USA) supplemented with 20% heat inactivated FCS (Lonza, Verviers, Belgium, selected batch), 2% pen-strep (Penicillin 10,000 UI/ml, Streptomycin 10,000 UI/ml, Lonza, Verviers, Belgium), and 2% l-glutamine (200 mM, Lonza, Verviers, Belgium) and activated with anti-CD3 and anti-CD28 linked with linker goat-anti-mouse antibody (BD Pharmingen, San Diego, CA, USA). 5 × 10^4^ PBMCs in 100 μl expansion medium were seeded per well in round-bottom 96-well plates (Nunc, Roskilde, Denmark) and incubated for 5 days. The immunosuppressive capacity of factors secreted by MSCs was evaluated by using the CM from the MSC culture conditions described earlier. One hundred microliters of CM of MSCs incubated with each of the SFs, except one of the OA donors were no SF was left, was added in triplicate to the PBMCs for 5 days. CM of MSCs without SF and unconditioned medium, identical to the medium used in the CM except for the fact that it had not been in contact with MSCs, were added in triplicate as a control. To correct for direct effects of the SF present in the CM on the PBMCs we added controls of medium not conditioned by MSCs with similar concentration of SF of each of the donors. At day four of incubation, ^3^H-thymidine (0.5 μCi/well; Perkin Elmer, Inc., San Jose, CA, USA) was added. At day five, after 16 h of incorporation of ^3^H-thymidine, PBMCs were harvested, and ^3^H-thymidine incorporation measured using a β-plate reader (Wallac 1450 MicroBeta TriLux Liquid Scintillation Counter and Luminometer, Perkin Elmer, Inc., San Jose, CA, USA).

### Data analysis

Statistical difference in gene expression by MSCs conditioned with SF(OA), SF(RA), and SF(control) was analyzed by using a mixed linear model in which condition(SF of OA, RA, or no joint pathology donors) was considered a fixed factor and the different SF donors for all conditions a random factor. Values for the genes IDO, TNFa, and TGFb were log-transformed to approach a normal distribution. Statistical differences of inhibitory capacity of the different CM was analyzed by using a mixed linear model in which condition (CM by MSCs incubated with OA, RA, or control SF) was considered a fixed factor, different donors a random factor and Sidak was used as adjustment for multiple comparisons. Inhibitory effects of CM with SF compared to SF only were explored by statistical analyses with the Wilcoxon signed ranks test. Data are presented as the mean ± standard deviation and 2.5–97.5 percentile. *P*-value of ≤0.05 was considered statistical significant; *P < 0.05, **P < 0.001. Analyses were performed using SPSS 17.0 Statistics (SPSS, Inc., Chicago, IL, USA).

## Results

### Effect of SF on gene expression of MSCs

**Figure 1:**
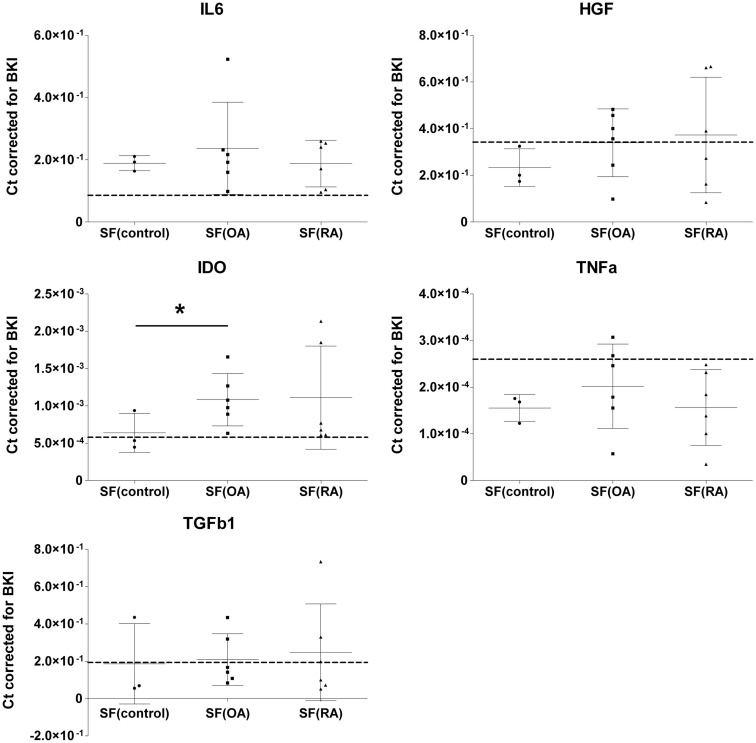
**Effect of different synovial fluids on gene expression of immunomodulatory factors by MSCs**. Gene expressions expressed in cycle thresholds (ct) normalized to BKI in every sample. MSCs were cultured in 20% SF of six OA and six RA donors and three donors without joint pathology. Dotted lines indicate the average gene expression in MSCs cultured in medium without SF. The data are presented as median scatterplots, each point represents an average of three measurements per donor (Mean ± SD). SF, synovial fluid; SF(control, OA, and RA) culture media of the MSCs supplemented with respectively non-pathological SF, OA SF, and RA SF. IDO, indoleamine 2,3-dioxygenase; IL-6, interleukin-6; TNFa, tumor necrosis factor-alpha; TGFb1, transforming growth factor-beta 1; HGF, hepatocyte growth factor; BKI, BestKeeper Index consisting of: GAPDH, UBC, and HPRT. *Expression in MSCs after culture in SF(OA) different from SF(control) by mixed linear test of these two conditions, *P* < 0.05.

To evaluate the effect of SF on mRNA expression of IL-6, HGF, IDO, TNFa, and TGFb1 by MSCs, MSCs were cultured in medium containing 20% SF of each of the 15 different donors. Medium with 1% ITS was used as SF free culture control and represented as a dotted line in Figure 1. Addition of SF significantly up-regulated IL-6 (2.43 ± 0.22-fold; *P* < 0.001) and IDO (1.72 ± 0.17-fold; *P* = 0.007) expression. There is a trend of down-regulation of TNFa albeit not significant. Gene expressions of HGF, TNFa, and TGFb1 were not significantly affected by SF compared to the SF free control (Figure 1).

Next, we explored the effect of three different types of SF separately. MSCs cultured in SF(OA) expressed IDO 1.69-fold (*P* = 0.048) higher than MSCs cultured in SF(control). For SF(RA) we also found an up-regulation in gene expression for IDO, albeit not significant which is probably caused by the large variation between the six different RA donors. No further significant differences in gene expression of IL-6, TNFa, TGFb1, and HGF were found between MSCs cultured in the three different SFs (Figure 1). IDO activity of MSCs was analyzed by an l-kynurenine assay on all different CM with SF corrected for l-kynurenine content in SF of that donor. No significant differences of IDO activity by MSCs cultured in SF of different donors were found (data not shown).

### Effect of conditioned medium on lymphocyte proliferation

**Figure 2:**
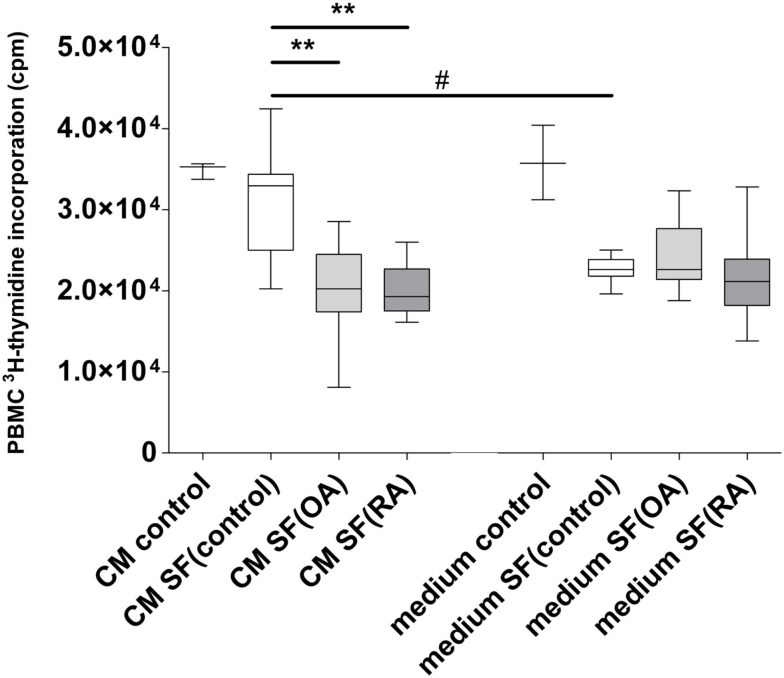
**Effect of conditioned medium of MSCs with different types of synovial fluid (SF) on proliferation of CD3/28 activated PBMCs**. Box-and-Whisker plot 2.5–97.5 percentile; *P < 0.05; **P < 0.001; ^#^*P* < 0.05 between CM and SF effect. CM, conditioned medium; SF, synovial fluid; (medium) culture medium with ITS; (CM control) MSC conditioned medium consisting of only culture medium with ITS; (Medium control) Only culture medium with ITS; SF (control) synovial fluid of donors without joint pathology; SF(OA) synovial fluid of patients with osteoarthritis; SF(RA) synovial fluid of patients with rheumatoid arthritis.

Conditioned medium harvested after culturing MSCs in 20% SF was used to analyze the effect of secreted factors of MSCs on the proliferation of CD3/CD28 activated PBMCs (Figure 2). The CM was mixed 1:1 with fresh medium and added to PBMCs. CM of MSCs without SF (CM control) did not influence PBMC proliferation. There was no difference in PBMC proliferation between CM of MSCs without SF and CM of MSCs incubated in SF(control). There was significantly more inhibition of PBMC proliferation by CM with SF(OA) compared to CM with SF(control; *P* < 0.001) and by CM with SF(RA) compared to CM with SF(control; *P* < 0.001).

To correct for direct effects of SF on PBMCs we added controls with unconditioned medium with SF. The SF appeared to inhibit PBMC proliferation, independent of disease state. A preliminary experiment with different concentrations of SF(OA) indicated that the effect of SF on lymphocyte proliferation is dose dependent (data not shown).

Conditioned medium of MSCs incubated in SF(control) caused significantly less inhibition of PBMC proliferation than unconditioned medium [medium that was not in contact with MSCs but contained SF(control)]. No significant differences in proliferation inhibition were found between OA and RA CM (Figure 2).

## Discussion

The aim of the study was to evaluate to what extent SF influences the immunomodulation of MSCs. This study indicates that SF can influence the expression of genes in MSCs that are involved in immunomodulation. Moreover, factors secreted by MSCs incubated with SF(OA and RA) inhibited the proliferation of activated lymphocytes significantly more than factors secreted by MSCs incubated without SF or with SF(control). This indicates that factors in diseased SF stimulate MSCs to secrete anti-inflammatory factors.

To our knowledge this is the first report on the effect of SF on the expression and secretion of immunomodulatory factors in MSCs. This information is important for the application of MSCs in joints of patients with joint diseases. Upon injection or implantation in the joint the MSCs will be exposed to SF. SF is known to contain a mix of factors secreted by the tissues of the joint. In the current study we investigated whether SF from non-diseased and OA and RA donors triggers MSCs to have an immunomodulatory effect. We demonstrated that SF(OA) can up-regulate MSC gene expression of IDO. In addition, MSCs treated with SF up-regulated expression of IL-6, a pleiotropic cytokine with pro-inflammatory functions, but also involved in regenerative processes and regulation of metabolism (Scheller et al., 2011). Which factor(s) secreted by MSCs cause the immunomodulatory effects cannot be concluded from our study and deserves further investigation.

We hypothesized that MSCs will be triggered by a catabolic environment in the joint to become immunomodulatory and that SF(RA) will induce large anti-inflammatory and SF(OA) will induce mild anti-inflammatory effects compared to SF(control). Our data could partly confirm this hypothesis. Diseased SF triggered MSCs to become immunomodulatory but we did not find any differences between the effects of SF(OA and RA) on gene expression of MSCs and PBMC proliferation. Whereas we assumed SF(control) would be immunological quiescent and diseased SF inflammatory, surprisingly we found inhibited lymphocyte proliferation by all SFs. This inhibition further increased by secreted factors of MSCs cultured with addition of SF(OA or RA), albeit non-significant. Surprisingly in the presence of SF(control), the inhibition of lymphocyte proliferation by SF was significantly reduced. This unexpected outcome suggests different effects of non-pathologic SF on excretion of factors by MSCs. Since the composition of healthy or diseased SF is not precisely known, it is difficult to explain the effects of SFs on MSCs and on PBMCs.

To provide a relatively clean way to study the effect of factors secreted by MSCs on lymphocyte proliferation, we used CM of MSCs exposed to SF. Different durations of exposure to SF and direct interactions between lymphocytes and MSCs in the presence of SF can play a role as well and this should be investigated in the future.

We here demonstrate that MSCs can be differently influenced by exposure to SF from diseased and non-pathological joints, but the effects were small compared to commonly used stimulation with TNFa and IFNg (Crisostomo et al., 2008; Hemeda et al., 2010; Hoogduijn et al., 2010). This might explain why resident MSCs in joints cannot prevent disease development; they might not be properly activated by the environment. It can also be regarded as somewhat disappointing in respect to the application of MSCs in the diseased joint since exposure to SF might not be sufficient to stimulate the healing activity of the MSCs.

Although it is unknown which factor in SF does stimulate MSCs, we performed ELISA on SFs. SF(RA) has a higher concentration of IL-6 compared to SF(OA; 7380 vs. 525.4 pg/ml; *P* = 0.009) and SF(control; 7380 vs. 22.4 pg/ml; *P* = 0.024), confirming previous reports (Kokebie et al., 2011). TNFa was measurable in only one OA donor and two RA donors and IFNg was measurable in only one OA and one RA SF donor (data not shown). Neither of these cytokines correlated with the effects of SF on MSCs or PBMCs but we can not exclude that other factors evoke an effect on MSCs. Moreover, *in vivo*, direct contact with MSCs and inflamed synovial tissue, immune cells in the synovium, or degenerated cartilage might, however, activate the MSCs. Finally, it should be noted that we have selected a limited number of immunomodulatory factors to evaluate the effect on MSCs and we cannot exclude that SF stimulates other processes in MSCs that can effect healing of the diseased joint.

Since the SFs were considered as redundant materials, ethical regulations preclude the availability of patient-specific information. It is very likely that the OA and RA patients used medication that might have influenced the compositions of the SFs. It has been demonstrated that analgesic drugs, NSAIDs, and DMARDs can change concentrations of immunomodulatory factors in SF (Bianchi et al., 2003, 2007; Alvarez-Soria et al., 2006). Use of different types of medication within donor groups could be a cause for the high variations within the groups.

Moreover, this explorative study was performed with SF of six OA donors, six RA donors, and three donors without joint pathology. To gain sufficient power the study should be repeated with larger numbers of pathological and non-pathological SFs. SF was used in a concentration of 20% for 48 h in analyses on MSCs. It remains unknown how MSCs will react on 100% SF over a longer period of time, which eventually will be the environment for MSCs when they are injected in a joint.

Although MSCs appear a promising therapy for degenerative joint diseases, the working mechanisms are not entirely clear. In animal studies it is possible to track MSCs injected in the joint. It was demonstrated that some of the injected MSCs stayed in the joint and adhered to the synovium or affected areas (Qi et al., 2011; Sato et al., 2012) from where they could exert a modulating effect and decrease the inflammatory or catabolic environment in diseased joints. The immunomodulatory capacity of MSCs can be useful for patients with OA and RA. Good therapeutic options for RA are already available, such as DMARDs and biologicals. However MSCs are capable of secreting many different factors, possibly for a prolonged time, which can influence many different mechanisms and are not restricted to one single target, unlike for example anti-TNFα. This explorative study shows that (1) SF can influence the expression of genes by MSCs involved in immunomodulation and (2) factors in CM by MSCs cultured with arthritic SF inhibit lymphocyte proliferation more than factors in CM by MSCs cultured without SF or with SF(control). These results warrant further research to examine the potential application of MSC therapy in arthritic joints.

## Conflict of Interest Statement

The authors declare that the research was conducted in the absence of any commercial or financial relationships that could be construed as a potential conflict of interest.
